# Parental age effect on the longevity and healthspan in *Drosophila melanogaster* and *Caenorhabditis elegans*

**DOI:** 10.18632/aging.205098

**Published:** 2023-11-01

**Authors:** Camille Lenzi, Alexis Piat, Pascal Schlich, Judith Ducau, Jean-Claude Bregliano, Hugo Aguilaniu, Anne Laurençon

**Affiliations:** 1 IM Projet, Neyron, France; 2 Caduceum, Lyon, France; 3 INRA, Centre des Sciences du Goût et de l’Alimentation (CSGA), Dijon, France; 4 IBDM, Parc Scientifique de Luminy, Marseille, France; 5 Instituto Serrapilheira, Rio de Janeiro, Brasil; 6 Institut de Genomique Fonctionnelle de Lyon, UMR5242, Universite Claude Bernard-Lyon 1, Ecole Normale Superieure de Lyon, Lyon, France

**Keywords:** life span, intergenerational plasticity, maternal effect, nematode, drosophila

## Abstract

Several studies have investigated the effect of parental age on biological parameters such as reproduction, lifespan, and health; however, the results have been inconclusive, largely due to inter-species variation and/or modest effect sizes. Here, we examined the effect of parental age on the lifespan, reproductive capacity, and locomotor activity of genetic isogenic lines of the nematode *Caenorhabditis elegans* and the fruit fly *Drosophila melanogaster*. We found that the progeny of successive generations of old parents had significantly shorter lifespans than the progeny of young parents in both species. Moreover, we investigated the fertility, fecundity, and locomotor activity of *C. elegans*. Interestingly, both the shorter lifespan and deteriorated healthspan of the progeny were significantly improved by switching to only one generation of younger parents. Collectively, these data demonstrate that the detrimental effect of older parental age on the longevity of the progeny can be reversed, suggesting the existence of a beneficial non–genetic mechanism.

## INTRODUCTION

Parental age at the time of reproduction inversely correlates with lifespan of the offspring in numerous species. In humans, it was found that earlier born brothers and sisters have longer lifespans than their later born siblings [1, 2; for review, 3]. Advanced parental age (generally considered >40 for women and >50 for men) also has a profound effect on the long-term health of the offspring, including increased risks of developing cancer, diabetes, and Alzheimer disease [4]. However, other studies have challenged these data. For example, in one study, the offspring of mothers >50 years of age had significantly better survival rates compared with the offspring of average age mothers (<33 years) [5]. Indeed, late pregnancy (women aged >45 years) has been associated with a rejuvenating effect that could also have an impact on the longevity of the offspring [6]. Given that the population of older individuals is increasing worldwide, these findings are of great relevance.

Studies with model organisms have played a pivotal role in understanding the cellular and molecular causes of aging, and will undoubtedly shed light on the biological mechanisms underlying the relationship between parental age and longevity. Setting the right conditions to study intergenerational links in model organisms will also help decipher molecular mechanisms at work in intergenerational healthspan inheritance.

Work by Lansing in the 1940’s and 1950’s evaluated the effects of parental age at the time of reproduction on the lifespan of the freshwater rotifer species *Philodina citrina* and *Euchlanis triquetra* [7, 8]. Using a series of orthoclones (successive generations having the same parental age), Lansing showed that older parental age had a negative cumulative effect on the offspring, leading to a progressively shortened lifespan in successive generations and, eventually, to clonal death. Notably, the short lifespan of an old orthoclone could be reversed by reproduction at a younger age, which increased the lifespan of subsequent generations and thus rescued the older orthoclones [7, 8]. Moreover, while orthoclones of older parental age (e.g., mating at 11 or 17 days of age) died out within 3 or 4 generations, Lansing showed that younger orthoclones (4 days) or reversed orthoclones could be maintained indefinitely [7, 8].

These studies established the notion that older mothers have shorter-lived offspring; a phenomenon known as the ‘Lansing effect’ [9]. Since then, the same effect has been reported for many species, including aphids (*Aphis nerii;* [10]), beetles (*Sitophilus oryzae;* [11]), butterflies (*Pieris brassicae;* [12]), and multiple fruit fly species (*Drosophila serrata;* [13]; *Drosophila mercatorum;* [14]; *Drosophila melanogaster*; [9]). Although the Lansing effect does not appear to be ubiquitous (e.g., cockroaches; *Nauphoeta cinerea*; [15]), the majority of studies support the notion that parental age negatively affects the fitness of the offspring and that parental age can have an important influence on population demographics [16]. In Drosophila, selection experiments where flies at old reproductive age (when roughly 40 to 80% of the population of flies had died) were bred have successfully selected long-lived and late fertility lines [17–20]. It is important to note that these experiments were performed with large population of genetically variable background animals.

Interestingly, while a number of studies have investigated the effects of advanced parental age on the lifespan of *D. melanogaster* ([16], reviewed by [21]), little work was published on the effect of parental age in the nematode *Caenorhabditis elegans*, a common model organism for aging studies [22–24]. Two recent articles have been published on the subject, addressing both the effect of old parental age on progeny longevity, and showing that one day old or three day old hermaphrodites produce progeny with identical life-span [25, 26]. Travers et al. also addressed the cumulative impact of advanced parental age on progeny, and showed that the ‘reversal effect’ observed by Lansing, was present in the nematode. We decided to investigate parental age impact on the lifespan of their progeny on selected genomes of flies and worms to gain insights on the molecular mechanisms at work.

**Figure 1:**
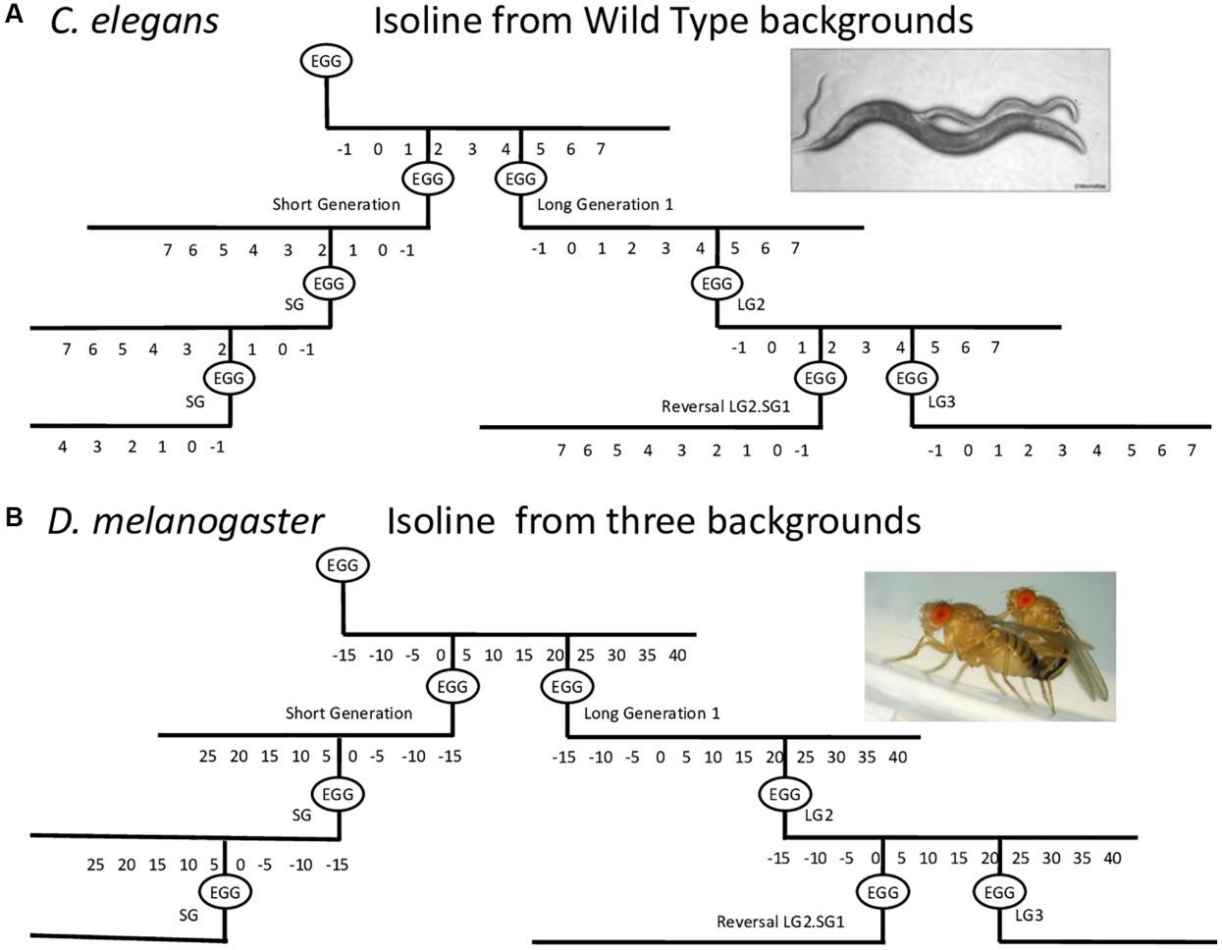
**Schematic of the procedure used to establish long and short generation lines with consistent parental age at reproduction.** Lines were established with short generation time (SG) or long generation time (LG) by maintaining the same parental age at reproduction for the next generation. For the reversal experiments, progeny of older parents were maintained for a certain number of long generations and then progeny were recovered at a younger age for several short generations. Using LG5.SG3 as an example, the progeny of the fifth long generation were recovered at a younger age for an additional three generations, and the progeny were then analyzed. The parental age for SG and LG lines was day 2 and day 4–5 of adulthood, respectively, for *C. elegans* (**A**), and day 2–6 and day 15–40 of adulthood, respectively, for *D. melanogaster* (**B**), depending on the genetic background.

We systematically investigated the impact of parental age on the lifespan, fecundity, fertility, and/or motility of the progeny of near isogenic lines (isolines: genetic inbred lines) maintained for generations with uniformly old parents (long generations or LG) or uniformly young parents (short generation or SG), or of ‘reversed’ lines which were switched from old to young parents (LG.SG, as initially observed by Lansing in rotifers, Figure 1). We show that parental age strongly influences the progeny lifespan in multiple genetically distinct strains of *C. elegans* and of *D. melanogaster*. Furthermore, we observe that, in *C. elegans*, the “reversed lines” harbor higher mean lifespan and healthspan than those of SG lines. These data reveal an age-dependent process that could help to identify the molecular mechanisms underlying the so-called ‘Lansing effect’.

## RESULTS

### Parental age affects the progeny lifespan in a reversible manner in *C. elegans*

To explore the effects of parental age on lifespan across multiple genetic backgrounds, we examined the lifespans of several isolines created from 13 wild-type strains of *C. elegans* (Supplementary Table 1 and [27]). From this analysis, we selected two long-lived strains (A, 22.5 ± 0.5 days and B, 22.4 ± 0.5 days) and two strains with intermediate lifespans (N2, 18.1 ± 0.2 days and C, 19.3 ± 0.3 days) for further work. Although short-lived strains were examined initially, their basal longevity showed considerable variability between experiments, and were therefore not further investigated.

**Figure 2:**
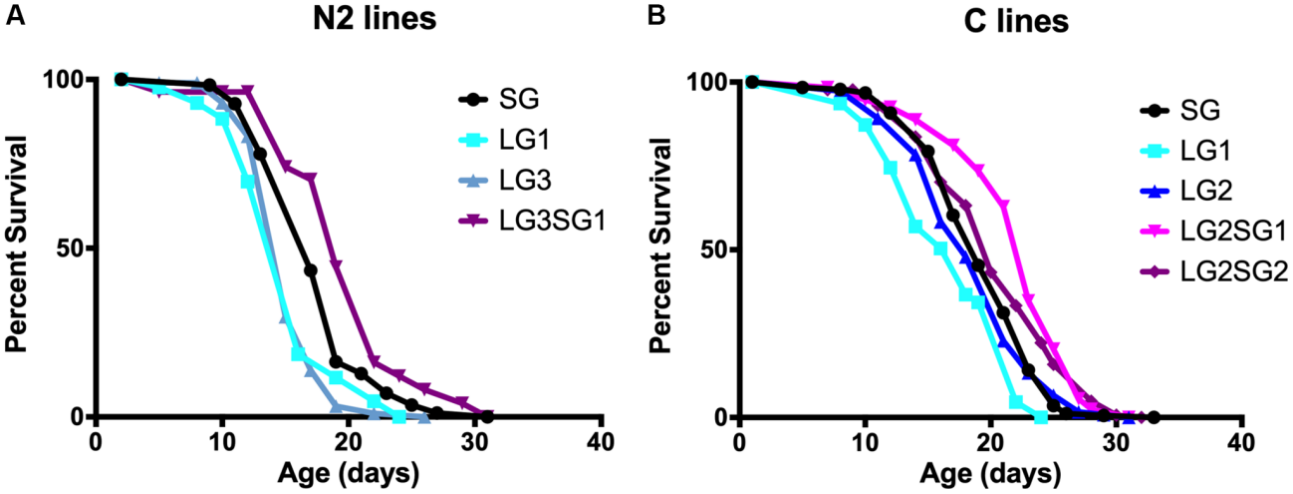
**Effect of parental age on the longevity of *C. elegans* progeny.** Lifespan analysis of *C. elegans* N2 (**A**) and C lines (**B**). SG animals were generation 5 compared to LG3 (**A**) and 4 compared to LG2 (**B**). *N* = 47–187 per line. See Table 2 for replicate experiments and statistical analysis.

The experimental setup for testing the parental age effect in *C. elegans* is shown in Figure 1A. We choose to maintain the lines with hermaphrodites and males, to enhance their reproductive capabilities. The average parental age for lines issued from young parents (SG) and older parents (LG) was 48 ± 4 h and 96 ± 12 h, respectively. We note that after five LG, the number of progeny was too small for statistical evaluation. Figure 2 shows representative data obtained with N2 and C lines. For both genetic backgrounds, the mean lifespan of the LG lines was significantly shorter than that of SG lines. For example, the mean lifespan of N2 hermaphrodites was 16.2 ± 0.4 days and 17.5 ± 0.4 days for LG3 and SG5, respectively (*p* = 0.001, Figure 2A), and the mean lifespan of C hermaphrodites was 16.7 ± 0.7 days and 19.3 ± 0.3 days for LG1 and SG2, respectively (*p* = 0.001, Figure 2B). Overall, the lifespans of LG lines were 4–16% shorter than those of SG lines (Table 1). We observed that the detrimental effect was observed after three successive generations, but not amplified. We conclude that there is no cumulative effect over generations and that one generation is sufficient to observe a significant loss of fitness.

**Table 1:** Replicate lifespan analysis for *C. elegans* lines.

**Strain**	**Genealogy**	**95% C.I. (days)**	**Mean LS ± SE (days)**	**Number of animals**	**Change (mean LS)**	***P*-value**	**Figure**
N2a	SG	17.1 ~ 18.2	17.65 ± 0.3	182			
N2a	LG1	14.1 ~ 16.5	15.35 ± 0.61	43	13	<0.001	
N2a	LG3	14.8 ~ 15.9	15.32 ± 0.28	102	13.2	<0.001	
N2a	LG3.SG1	17.8 ~ 21.7	19.74 ± 0.99	27	−11.8	0.03	
N2c	SG	16.6 ~ 17.7	17.49 ± 0.39	166			2A
N2c	LG1	16 ~ 17.4	16.68 ± 0.36	160	4.6	0.01	2A
N2c	LG3	15.4 ~ 17.1	16.25 ± 0.43	113	7.1	0.001	2A
N2c	LG3.SG1	19.2 ~ 20.4	19.79 ± 0.29	127	−13.1	0.005	2A
N2c	LG2	15.4 ~ 17.4	16.41 ± 0.5	141			
N2c	LG2.SG1	18.1 ~ 19.8	18.99 ± 0.44	163	−15.7	<0.001	
A	SG	21.6 ~ 23.7	22.68 ± 0.54	257			
A	LG1	19.7 ~ 21.6	20.67 ± 0.49	244	8.9	0.002	
A	SG	27.2 ~ 30.6	28.94 ± 0.86	169			
A	LG1	22.9 ~ 25.4	24.16 ± 0.62	165	16.5	0.003	
A	SG	25.8 ~ 28.8	27.30 ± 0.77	96			
A	LG2	21.9 ~ 25.8	23.88 ± 0.99	90	12.5	<0.001	
A	LG2	21.5 ~ 24.4	22.99 ± 0.73	116			
A	LG2.SG1	23.5 ~ 26.2	24.85 ± 0.69	130	−8.1	<0.001	
C	SG	18.6 ~ 19.9	19.27 ± 0.33	184			2B
C	LG1	15.4 ~ 18.1	16.74 ± 0.7	47	13.1	<0.001	2B
C	LG2	17.8 ~ 19.4	18.64 ± 0.4	148	3.3	0.45	2B
C	LG2.SG1	21.2 ~ 22.6	21.94 ± 0.37	187	−13.9	<0.001	2B
C	LG2.SG2	19.6 ~ 21.2	20.38 ± 0.41	173	−5.8	0.004	2B
C	LG2	14.1 ~ 16.3	15.2 ± 0.58	80			
C	LG2.SG1	19.5 ~ 21.3	20.39 ± 0.46	122	−34.1	<0.001	

Data used to construct Figure 2 are indicated. LS = lifespan; change mean LS = percentage difference compared with the SG lifespan (negative value indicates an increase in mean lifespan). For experiments where SG was not evaluated, the *P*-value compared the two samples presented. *P*-values were calculated using the log-rank method.

To test the reversibility of the parental age effect in *C. elegans*, we maintained animals for 2 (C) or 3 (N2) LG and then switched them to 1 or 2 SG. The progeny was collected, and longevity measured. Consistent with a reversible ‘Lansing effect’, we found that the resulting lifespan of N2 hermaphrodite reversal lines was significantly longer than the progeny of SG animals (LG3.SG1, 19.7 ± 1.0 days and SG4, 17.7 ± 0.3 days, *p* = 0.03; Figure 2A). Finally, experiments with C lines (Figure 2B) or A lines (Table 1) confirmed that late reproduction significantly shortened the mean lifespan of the progeny, and this can be reversed upon switching to younger age at reproduction (Table 1). Taken together, these data indicate that parental age at reproduction significantly affects the progeny lifespan in *C. elegans*, and that the effect can be reversed when the progeny of older parents are allowed to reproduce at a young age.

### Advanced age at reproduction impacts fecundity of progeny in *C. elegans*

In the designed protocol, both hermaphrodites and males were included (see material and methods for further details). It is notorious that male addition increases by at least two-fold the number of progeny produced by hermaphrodites. During the conduct of these experiments, we observed that the different LG lines gave rise to few progeny. Therefore, we asked whether parental age effect influences the fecundity of progeny by investigating single hermaphrodite in a longitudinal assay from SG, LG, and LG.SG lines evolving in parallel. We choose to study both the N2 and the A lines, because they have different reproductive profile (Supplementary Table 2). Under these experimental conditions, we observed that fertilization of hermaphrodites by males was not 100% efficient (i.e., the progeny was not always 50% males) and a high occurrence of intrauterine larval development, leading to the destruction of the female by the larvae or juveniles (matricidal hatching, Supplementary Table 2). This is a known result of long-term mating with males.

**Figure 3:**
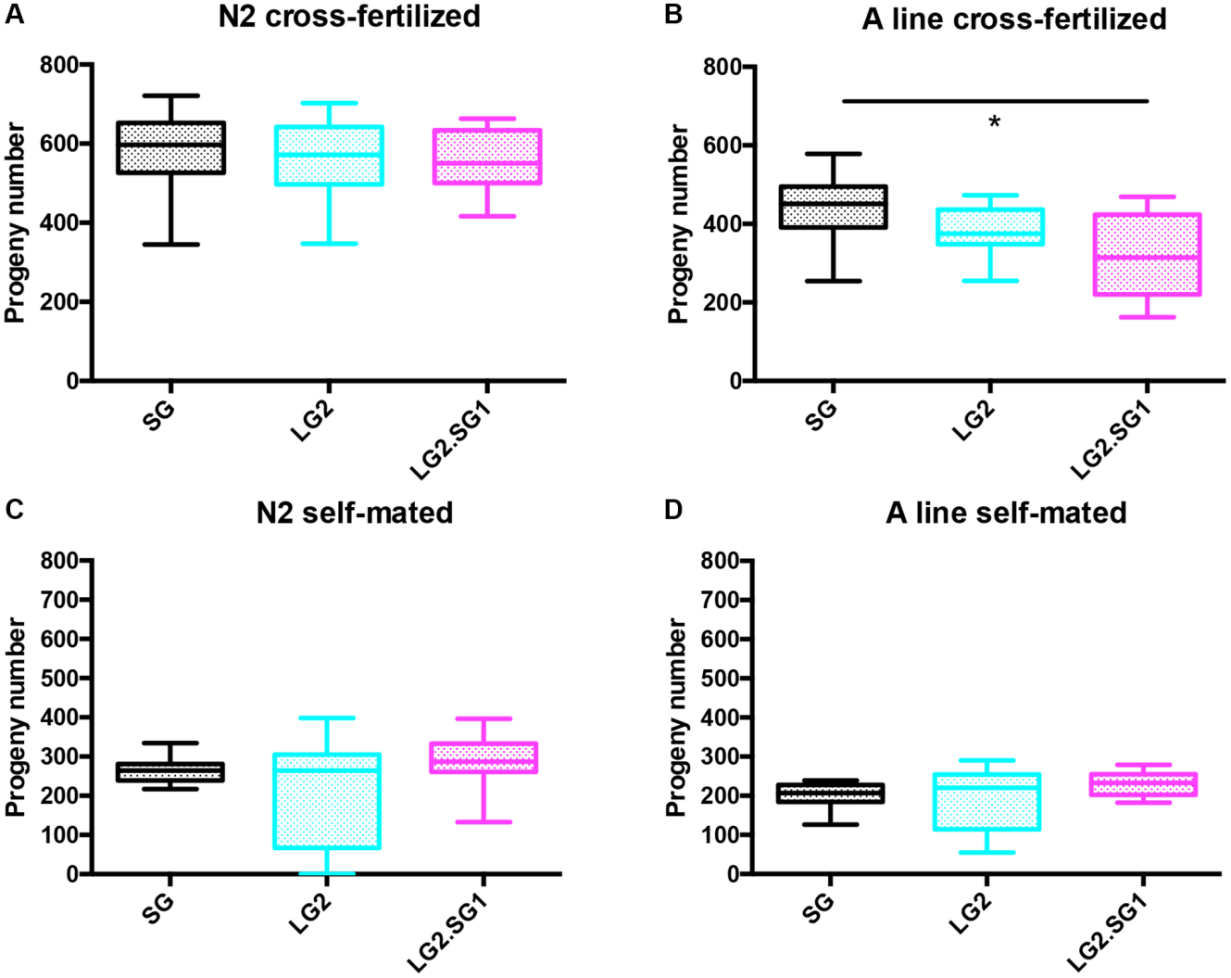
**Effect of parental age on the fecundity of *C. elegans* progeny.** (**A**, **B**) Total progeny of cross-fertilized hermaphrodites produced between 36 to 156 h of adulthood (hermaphrodites were sorted based on fluorescent sperm transfer from males after a 12 h copulation window). Mean ± SD, n > 30. (**C**, **D**) Total progeny of self-fertilized hermaphrodites produced between 0 to 156 h of adulthood. Mean ± SD, *n* > 18. ^*^p < 0.05, ^**^p < 0.001 by one-way ANOVA (Kruskal-Wallis test).

To circumvent these technical difficulties, we tested the effects of male fertilization on the fecundity (number of progeny produced) and fertility (embryo hatching rate) of hermaphrodites. To this end, we compared hermaphrodites that were self-fertilized or fecundated by males. To minimize the time of exposure to males (leading to matricidal hatching), we identified hermaphrodites that had copulated within a 12 h time windows using a sex-specific fluorescent Mitotracker mating assay [28–30]. This method enabled the selection and isolation of individual cross-fertilized hermaphrodites. As expected, hermaphrodites fecundated by males produced at least twice the number of progeny than self-fertilized hermaphrodites (compare Figure 3A–3D). For example, self-fertilized N2 SG4 animals produced 239.2 ± 25 progeny over 36–156 h of adulthood, while cross-fertilized hermaphrodites SG4 produced 589 ± 87 progeny over the same time-frame, and similar results were obtained with the A lines. We also measured the rate of egg hatching in these experiments and found that self-fertilized animals showed reduced fertility after 96 h of age, as expected due to lack of sperm (Supplementary Figure 1).

We first found that the number of progeny from LG2.SG1 was significantly reduced for LG2.SG1 A lines (Figure 3B and Supplementary Table 2). In both N2 and A backgrounds, the progeny of cross-fertilized LG2.SG1 reversal lines showed reduced fertility compared with the progeny of mated SG4 lines, especially in the second part of their reproductive lifespan (age >96 h, statistically significant for both backgrounds, Supplementary Figure 1). Overall, male fecundated hermaphrodites exhibited subtle differences in fecundity between isolated lines, and the reversal lines progeny were the most negatively affected both in term of fertility and fecundity.

For self-fertilized animals, the impact of the parental age effect was the same on both genetic backgrounds. LG2.SG1 lines consistently gave rise to more progeny than either the self-fertilized SG or LG lines. Brood size for SG4 and LG2.SG1 lines were 203 ± 29 and 222 ± 30 for A lines and 264 ± 27 and 291 ± 57 for N2 (Figure 3C, 3D and Supplementary Table 2). Hermaphrodites from LG2 lines presented a highly variable level of fecundity, for both genetic backgrounds.

Collectively, these results demonstrate that parental age has a subtle effect on the fertility and fecundity of progeny in *C. elegans*. In general, animals derived from older parents exhibit highly variable fecundity. In reversal lines, self-fertilized animals tend to produce a larger number of progeny. Additionally, we observed that the progeny of the reversal lines hatched at a lower rate when fecundated by males, especially in the second phase of their reproductive span. We also observed that the brood span of these animals is reduced. In the future, it will be interesting to examine the quality and number of oocytes and sperm in these animals.

### Parental age influences locomotor behavior in *C. elegans* progeny

**Figure 4:**
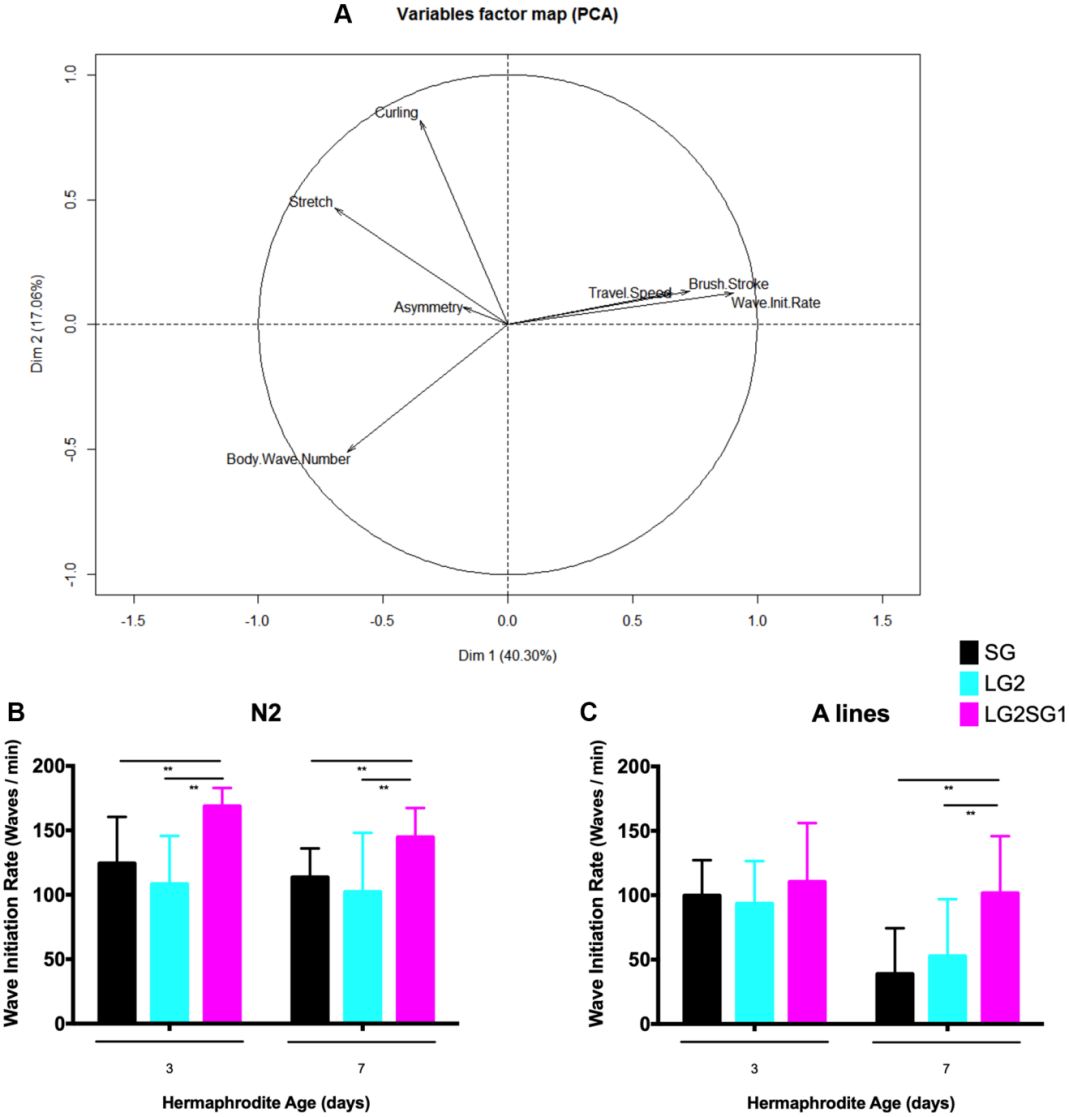
**Effect of parental age on the motility of their progeny in *C. elegans*.** (**A**) Principal component analysis of the swimming behavior of N2 *C. elegans.* Progeny from SG4, LG2, LG2.SG1 lines were evaluated on day 3 and day 7 of adulthood. Eight motility measures were calculated from movies of two independent experiments. PC1 was maximally discriminant for the three categories of worms (SG4, LG2, LG2.SG1), with 80% composed of wave initiation rates. This axis also discriminates between the two time points (ages 3 and 7 days). (**B**, **C**) Wave initiation rates (waves/min) for N2 (**B**) and A (**C**) lines. Mean ± SD for *n* > 28 per condition. ^**^p < 0.01 by two-way ANOVA.

To assess whether parental age affects other aspects of the physiology of *C. elegans*, we examined locomotion as a marker of overall health. For this, we used the CeLesT computational program [31, 32] to measure the animals’ capacity to swim continuously over a defined period of time. Consistent with previous data [31], we observed age associated alterations in several swimming parameters. PCA analysis of data from N2 lines showed that travel speed, brush stroke, and wave initiation rate decreased with age, while asymmetry and stretch and curling rates increased (Figure 4A). Of the parameters analyzed, we identified wave initiation rate as the best discriminant to investigate the effects of generational history (SG4, LG2, and LG2.SG1) on locomotor behavior. We found little to no difference in the wave initiation rate between progeny derived from cross-fertilized or self-fertilized hermaphrodites for the different lines analyzed (data not shown). However, we consistently observed significantly higher wave initiation rates in the reversal lines compared with either the SG or LG lines. For example, 3-day-old N2 LG2.SG1 hermaphrodites showed a rate of 168 ± 14 waves/min compared with 124 ± 36 and 108 ± 37 waves/min for SG4 and LG2 animals, respectively (Figure 4B). Similarly, on the A background, 7-day-old LG2.SG1 animals showed a mean wave initiation rate of 101 ± 44 compared with 38 ± 35 and 52 ± 44 waves/min for SG4 and LG2 animals (Figure 4C). However, there was no significant difference between the wave initiation rates of SG and LG animals of either genetic background (Figure 4B, 4C).

### Advanced parental age shortens the lifespan of *D. melanogaster*

**Figure 5:**
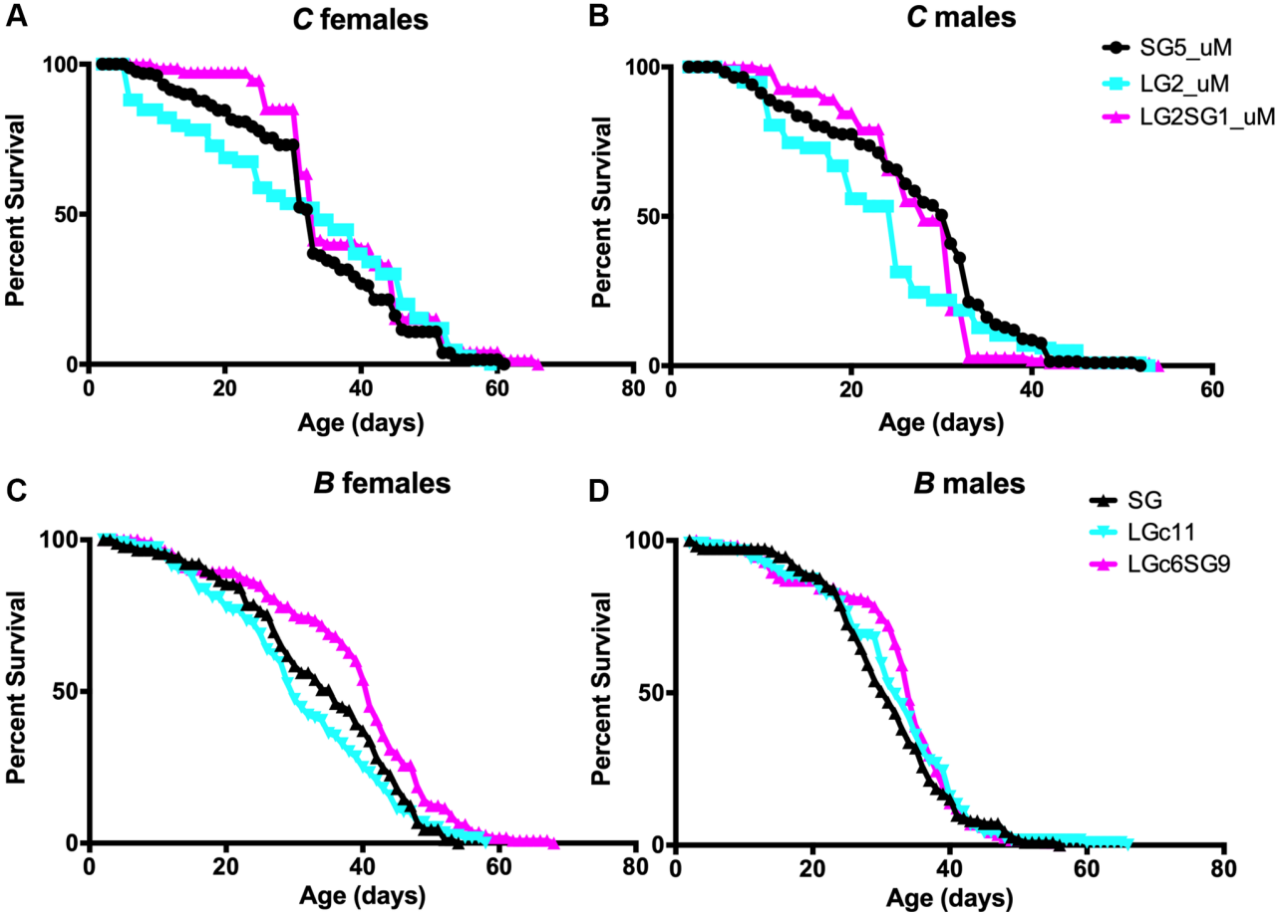
**Effect of parental age on the longevity of *D. melanogaster* progeny.** (**A**–**D**) Lifespan analysis of female (**A**, **C**) and male (**B**, **D**) for *C* lines (**A**, **B**) and *B* lines (**C**, **D**) of flies. Lifespan analysis of separated sexes (uM) for *C* lines, which genetics background is related to stock *B*, confirms that the observation with mixed sexes is similar when observing parental age effect. See Table 1 for replicate experiments and statistical analysis.

We investigated the impact of parental age on the lifespan of *D. melanogaster* using two strains here named *A* and *B*, which were examined as part of a larger study (see materials and methods, [33, 34]) with the experimental setup shown in Figure 1B. The average parental age was 4 ± 2 days for the SG isolines and 40 ± 2 days and 20 ± 2 days, respectively for *A* and *B*, for the LG isolines (Figure 1). In multiple experiments, the lifespan of the LG lines from A lines was found to be shorter than that of the SG lines by 16% for the females (*p* = 0.007; LGb39 34.8 ± 1.5 days, SG112 41.4 ± 1.4 days) and 9% for the males (*p* = 0.03; LGb39 42.9 ± 1.3 days, SG112 47.0 ± 1.1 days) (Table 2, Supplementary Figure 2A–2D, see experimental procedures for nomenclature). Similarly, the lifespan of *B* LG lines was shorter than that of the SG lines for females. This effect was not consistently observed for males in this genetic background. For example, in the experiment shown in Figure 5C, 5D, the mean lifespans were 31.1 ± 1.1 days and 33.8 ± 1.3 days for LGc11 and SG22 females, respectively (*p* = 0.001) and 31.5 ± 1.0 days and 30.8 ± 1.0 days for LGc11 and SG22 males, respectively (not significant). Overall, the results of multiple experiments for five independent lines issued from A and B strains indicated that the mean lifespans were 10–30% shorter for the LG than the corresponding SG lines (Table 2).

**Table 2:** Replicate lifespan analysis for *D. melanogaster* lines.

**Strain**	**Genealogy**	**Gender**	**95% C.I. (days)**	**Mean LS ± SE**	**Number of animals**	**Change mean LS**	***P*-value**	**Figure**
C	SG5	FuM	30.9 ~ 35.0	32.98 ± 1.05	163			5A
C	LG2	FuM	28.9 ~ 34.1	31.55 ± 1.32	151	4.3	0.44	5A
C	LG2.SG1	FuM	34.8 ~ 39.6	37.23 ± 1.23	121	−12.8	0.04	5A
C	SG5	MuM	26.0 ~ 28.7	27.39 ± 0.68	233			5B
C	LG2	MuM	21.9 ~ 25.8	23.90 ± 1.00	120	12.7	0.05	5B
C	LG2.SG1	MuM	25.7 ~ 28.3	27.04 ± 0.66	128	1.3	0.02	5B
B	SG22	F	31.3 ~ 36.2	33.8 ± 1.26	89			5C
B	LGc11	F	28.8 ~ 33.3	31.06 ± 1.14	116	8.1	0.001	5C
B	LGc6.SG9	F	36.2 ~ 40.8	38.55 ± 1.18	113	−14	0.29	5C
B	SG22	M	29.1 ~ 32.6	30.82 ± 0.9	113			5D
B	LGc11	M	29.6 ~ 33.4	31.54 ± 0.97	119	−2	0.38	5D
B	LGc6.SG9	M	30.9 ~ 34.5	32.67 ± 0.92	115	−6	0.15	5D
A	SG112	F	38.7 ~ 44	41.37 ± 1.36	112			Supp 2C
A	LGb39	F	32 ~ 37.7	34.83 ± 1.45	108	15.8	0.007	Supp 2C
A	LGb33.SG12	F	38.3 ~ 42.2	40.31 ± 0.99	114	2.6	0.01	Supp 2C
A	SG112	M	44.8 ~ 49.2	47.02 ± 1.13	107			Supp 2D
A	LGb39	M	40.3 ~ 45.4	42.87 ± 1.29	100	8.8	0.03	Supp 2D
A	LGb33.SG12	M	46.6 ~ 50.2	48.44 ± 0.93	107	−3	0.89	Supp 2D
A	SG98	F	42.6 ~ 47.7	45.14 ± 1.3	111			Supp 2A
A	LGa34	F	30.8 ~ 34.3	32.57 ± 0.9	110	27.8	<0.001	Supp 2A
A	SG98	M	46.6 ~ 51.4	49.02 ± 1.24	110			Supp 2B
A	LGa34	M	36.8 ~ 39.7	38.29 ± 0.75	107	21.9	<0.001	Supp 2B
B	SG	F	32.7 ~ 39.8	36.25 ± 1.8	110			Supp 3C
B	LGb2	F	29.7 ~ 33.1	31.4 ± 0.86	117	13.4	<0.001	Supp 3C
B	LGa4	F	31.6 ~ 35.5	33.59 ± 0.99	111	7.3	<0.001	Supp 3C
B	SG	M	24.4 ~ 30.1	27.25 ± 1.44	113			Supp 3D
B	LGb2	M	28.4 ~ 31.5	29.98 ± 0.81	114	−10	0.34	Supp 3D
B	LGa4	M	32.5 ~ 35.2	38.84 ± 0.69	113	−42	0.27	Supp 3D
B	SG22	F	37.6 ~ 43.2	40.43 ± 1.44	120			Supp 3A
B	LGa11	F	27.7 ~ 34	30.86 ± 1.61	118	23.7	<0.001	Supp 3A
B	SG22	M	29.8 ~ 34.3	32.08 ± 1.17	120			Supp 3B
B	LGa11	M	23.9 ~ 28.8	26.34 ± 1.26	118	17.9	0.002	Supp 3B
B	SG22 x LGc11	F	32.4 ~ 38.1	35.3 ± 1.46	96			Supp 3E
B	LGc11x SG22	F	30.8 ~ 35.6	33.23 ± 1.25	115	5.9	0.11	Supp 3E
B	SG22 x LGc11	M	30.8 ~ 35.2	33 ± 1.12	96			Supp 3F
B	LGc11x SG22	M	28.6 ~ 32.2	30.43 ± 0.91	118	8.1	0.008	Supp 3F

Data used to construct Figure 5 and supplementary figures are indicated. LS = lifespan; change mean LS = percentage difference compared with the SG lifespan (negative value indicates an increase in mean lifespan). *P*-values were calculated using the log-rank method.

During these experiments, there was a considerable number of generations that separated the SG and LG lines. We aimed to investigate whether a shorter number of successive LG could have an impact. Our observations on *B* strain revealed a decrease in the mean lifespan of the LG lines after 2, 4, and 11 long generations in each experiment (Table 2, Supplementary Figure 3A–3D). Furthermore, the life span experiments were conducted by mixing sexes throughout the duration of experiment. We thus set out to reproduce these observations using *C* line, which share common ancestors with *B* lines, and probe longevity with separated sexes (unmixed sexes). Similar trends were observed in both sexes with a significant difference in males (*p* = 0.05; LG2 23.2 ± 1 days, SG5 27.4 ± 0.7 days) and a non-significant difference in females (LG2 31.5 ± 1.3 days, SG5 32.9 ± 1 days) (Figure 5A, 5B). We thus observed that the detrimental effect was observed after two successive generations, but we did not observe any amplification of this effect over subsequent generations. We conclude that there is no cumulative effect over generations in *Drosophila melanogaster*, as well as in the nematode.

### The heritable parental age effect in *Drosophila* is reversible

The Lansing effect is reversible and therefore supports the notion that shortening of lifespan with increasing parental age is independent of nuclear genetic changes in the progeny. To test whether this is also true in our experiments in *D. melanogaster*, we recovered the progeny of *A* and *B* lines maintained for multiple LG (33 generations with parental age 40 ± 2 days (*A*) and six generations with parental age 20 ± 2 days (*B*) in Figure 5A, 5B) followed by a switch to younger reproductive age (4 ± 2 days) for 12 (*A*) or 9 (*B*) SG generations (Figure 5A, 5B, Supplementary Figure 2C, 2D). We found that the lifespan of the *A* LGb33.SG12 males was significantly longer than that of the LGb39 line (48.4 ± 0.9 days and 42.8 ± 1.3 days, respectively, *p* = 0.01) and was virtually the same as the SG112 line (47 ± 1.1 days; 3% difference) (Table 2). For *A* females, the difference in lifespan between LGb39 and LGb33.SG12 was robust in the first part of the curve only (days 1–40 in Figure 2A; Wilcoxon test, *p* = 0.006). Although the longevity of *A* females SG112 and LGb33.SG12 lines was similar (41.4 ± 1.4 days and 40.3 ± 1.0 days, respectively; 2% difference), the tail distribution distinguishes the two populations (Supplementary Figure 2C, 2D).

The reversibility of the parental age effect was also observed in the *B* background; however, the mean lifespan difference between animals maintained on LG and reversed from LG to SG was significant for females (LGc11, 31.1 ± 1.1 days and LGc6.SG9, 38.5 ± 1.2 days; *p* < 0.001) but not for males (LGc11, 31.5 ± 1.0 days and LGc6.SG9 32.7 ± 0.9 days; Figure 5A, 5B and Table 2). The mean lifespan of *B* LGc6.SG9 females was not significantly different from that of the SG22 line (38.5 ± 1.2 days and 33.8 ± 1.3 days, respectively; *p* = 0.29) (Figure 5C, 5D and Table 2). Interestingly, we were able to reproduce the reversal effect in one short generation: both females and males of *C* line displayed similar or longer lifespans compared to the SG lines (Figure 5A, 5B).

These observations are consistent with the possibility that the parental age effect is maternally driven because we observed that hybrids between SG and LG lines had different lifespans depending on parental origin. The mean lifespan of the progeny of LG11 females (33.2 ± 1.2 and 30.4 ± 0.9 days for female and male progeny, respectively) was shorter than that of the reciprocal hybrids derived from SG22 females (35.3 ± 1.5 and 33.0 ± 1.1 days for females and males, respectively; Supplementary Figure 3E, 3F), but the differences were significant only for males (*p* = 0.008). This finding suggests that maternal origin is determinant for the lifespan of the progeny in *D. melanogaster*, consistent with previously published observations [21, 9].

## DISCUSSION

### Effect of parental age on progeny physiology

In this study, we confirmed that parental age affects the longevity of *D. melanogaster* and *C. elegans* and that this effect is both transmissible over generations and reversible. Animals derived from young parents, which is the standard method of propagation of animals in the laboratory, have normal brood sizes and lifespans of stable durations over multiple generations. In contrast, animals born to older parents have shorter lifespans. In *D. melanogaster*, the magnitude of the effect on lifespan ranged from 10% to 30%. It will be interesting to determine the cause of this variation. This degree of variation in lifespan is similar to that previously reported with *D. melanogaster* [21, 9]. In the case of *C. elegans*, we observed a decrease in mean lifespan of ~10% in the LG lines, and we could not maintain LG lines more than five successive generations. A small but significant effect of parental age on lifespan was already observed by Klass [24], who, also used LG lines maintained at parental age 5–6 days, albeit without mating. Our findings contrast with other studies in which lines derived from day-3 adults showed no significant shortening of lifespan compared with lines generated with day-1 adult hermaphrodites [25, 26, M. Hansen personal communication].

We also found that the effects of parental age on fecundity in *C. elegans* progeny are affected by the propagation strategy (i.e., self-fertilization vs. mating with males). Beguet et al. reported a significant decrease in the fecundity of *C. elegans* progeny from older compared with younger self-fertilized parents, and the effect was reversible [22, 23]. Although we observed a similar trend with self-fertilized animals, the difference between the number of progeny in LG and SG lines was not statistically significant. However, LG.SG lines produced a larger number of progeny than either LG or SG lines. Interestingly, the opposite phenotype was observed for the reversal lines of cross-fertilized hermaphrodites in two backgrounds, i.e., the number of progeny of LG.SG lines was smaller than that of the SG or LG lines. We speculate that this could be linked to reduced fertility observed in the second part of the reproductive life of the LG.SG lines compared with the SG and LG lines. This result may reflect oocyte frailty that can only be detected when reproduction is achieved when hermaphrodites mate with males which allows them to produce large quantity of oocytes. A more detailed analysis of the physiology of gonads in these lines will help understand the mechanisms at stake.

We also found that reproduction was delayed in the offspring of old *C. elegans* compared with young parents (beginning on day 3 and day 1, respectively), which was also observed in rotifers [8]. This observation suggests that parental age may shift the timing of reproductive maturity. Older nematodes have been shown to lay larger eggs that develop more slowly and lead to progeny with larger body sizes [25, 26]. This is also observed in mites [35]. Although developmental rate could be an important parameter through which parental age influences lifespan, in our hands, animals derived from older parents tended to develop faster than the progeny of young parents (data not shown).

Another factor that may affect the lifespan of *C. elegans* is the density at which the parents are grown. For example, a difference in mean lifespan of up to 4 days has been reported between animals raised from 1 egg/plate compared with 50 eggs/plate [36]. In our experimental setting, fewer animals were generated from plates of eggs derived from LG parents compared with SG parents. This may have influenced the lifespans of progeny derived from LG vs. SG parents. However, we believe that such an effect would lead to an underestimation of mean lifespan differences in the progeny of SG vs. LG parents, not an overestimation.

### Reversal of the parental age effect on progeny lifespan and healthspan

Although many studies of various species have found that advancing parental age shortens progeny lifespan, the reversibility of this effect has not attracted a similar degree of attention [37]. Here, we found that a switch from LG to SG parents restored lifespan to values observed in SG progeny in both *D. melanogaster* and *C. elegans.* In fact, longevity and fecundity of self-fertilized animals were both enhanced in *C. elegans* reversed lines compared with SG lines. Interestingly, this reversal effect was also observed with other health span-associated parameters, such as swimming capacity. Here too, the reversal effect on wave initiation rate was observed independently of the *C. elegans* wild-type background.

A single generation of reversal is enough to restore longevity and healthspan in the nematode. We thus think that the impact of late reproduction that we describe is not mediated by the selection of genotypic variation. Moreover, genetic selection for old reproductive animals has been proven to lead to long lived lines while we describe a depressive effect on longevity in the LG lines. These observations are consistent with the idea that an epigenetic mechanism is at work in the physiological changes we observed.

The epigenetic mechanisms underlying the parental age effect observed in this study are unknown. In the nematode, histone modifications have been investigated as a central mechanism of transgenerational effects on longevity [38, 39]. Similarly, small RNAs have been linked to several transgenerational phenotypes, including longevity, especially under conditions of nutritional deprivation and fluctuating environmental temperature [40–42]. It will be interesting to determine whether the SG, LG, and LG.SG animals studied here differ in chromatin structure and regulatory elements that could influence gene expression and/or in the accumulation of factors in the germ line.

The accumulation and transmission of aging factors have been described in the budding yeast *Saccharomyces cerevisiae* [43, 44]. In *C. elegans* and *D. melanogaster*, the germline (particularly oocytes) is thought to be protected from the accumulation of factors having deleterious effects on the aging process; however, germline also shows signs of decreased molecular homeostasis and increased damage over time [45, 46]. In the copepod *Acartia tonsa* (an aquatic microinvertebrate), an accumulation of carbonylated proteins was observed in the offspring of older mothers compared with younger mothers, suggesting that oxidative damage could be transmitted through reproduction [47].

Further studies will be needed to understand the molecular basis of the transgenerational phenotypes described here. Importantly, the magnitude of the healthspan differences between the progeny of SG, LG, and LG.SG lines of *C. elegans* are sufficiently robust to enable investigations at the molecular level. As described above, several non-exclusive mechanisms could be responsible for the parental age effect.

## MATERIALS AND METHODS

### Strains and cultures

*C. elegans*: Strains were inbred by individual self-fertilization for at least ten generations to generate isogenic lines from 13 different genetic backgrounds (See Supplementary Table 1). Six independent isolines on each background (designated a–f) were generated and frozen. All worms were maintained using standard protocols on Nematode Growth Medium (NGM) with *E. coli* strain OP50 at 20°C (Sulston and Hodgkin 1988) for generation of isogenic lines and for experiments. During the experiments, hermaphrodites were mated with males; the age of the animals at reproduction was 96 ± 12 h of adulthood for the LG lines (experiments were also performed at age 120 ± 12 h; data not shown) and 48 ± 2 h for the SG lines. Background strains were obtained from the Caenorhabditis Genetics Center, which is funded by National Institutes of Health Office of Research Infrastructure Programs (P40 OD010440).

*D. melanogaster*: Most of the experiments conducted in drosophila were examined as part of a larger study of flies with differing levels of reactivity, a phenomenon that influences I element retrotransposons during hybrid dysgenesis crosses [33]. However, this is not discussed here because we have found no direct correlation between strain reactivity level and longevity. The *D. melanogaster* stocks used were A: *e*stM bearing ebony mutation, and B: *Paris* bearing cinnabar mutation. B strain was selected for several generations by successive individual crosses to lead to PF2 [32]. Another line was more recently derived from *Paris* stocks and studied here as C. Flies were reared on axenic food (Agar, Yeast, Corn flour and fungicide paraben) in uncrowded conditions (30–50 animals/vial containing 6 ml of food) [48]. Temperature (20 ± 0.5°C) and fly age were carefully controlled. Flies were handled at room temperature under ether anesthesia. The average age of the SG lines was 4 ± 2 days. The average age of the *A* and *B* mothers was 40 ± 2 and 20 ± 2 days, respectively, for the LG lines. For C stock the average age of the females for the LG lines was 14 ± 2 days. A total of 30–60 fly pairs were used for each generation (more for LG lines to compensate for greater mortality).

Lines were established with short generation time (SG) or long generation time (LG) by maintaining the same parental age at reproduction for the next generation. The number of generations separating the two lines are indicated after SG or LG. If a letter is added after LG, it differentiates the different lines derived at different time point from the SG line it is compared to. For the reversal experiments, progeny of older parents was maintained for a certain number of long generations and then progeny was recovered at a younger age for several short generations. Using LG5.SG3 as an example, the progeny of the fifth long generation were recovered at a younger age for an additional three generations, and the progeny were then analyzed.

### Lifespan analysis

*C. elegans*: Lifespan assays were conducted at 20°C and only hermaphrodites were analyzed. Worms were synchronized by placing adults on NGM plates seeded with OP50, allowing them to lay eggs for 2 h, and then removing the adults. The eggs were then grown to the L4 stage and transferred to OP50-seeded NGM plates containing 15 μM 5-fluorouracil. The day of transfer was scored as day 0 of the experiment. For these experiments, OP50 were UV-irradiated for 10 min (6 J/cm^2^) using a UV Stratalinker 2400. In our hands, this treatment completely inhibits bacterial growth. Animals that failed to respond to repeated prods with a platinum pick were scored as dead, and animals were censored if they crawled off the plate or died from vulval bursting. For each lifespan assay, at least 120 worms (20 worms per plate) were analyzed.

*D. melanogaster*: Animals were synchronized by recovering eggs after an 8-h laying period on the day of parental transfer. Adult flies were collected at eclosion (day 0) and were placed in groups of 20 males plus 20 females in food vials (6 vials per condition). For the unmixed sex conditions, 20 animals were pooled 20 per vials. Flies from each vial were transferred daily and dead flies were scored but not replaced. Assays shown on a single graph were performed in parallel on the same days.

### Fertility, fecundity, and development time

*C. elegans*: Fecundity (number of progeny produced) and fertility (embryo hatching rate) were determined by longitudinal assays. L4-stage hermaphrodites were placed on dishes (1 per dish) and transferred to a fresh dish every 12 h (or 6 h during the period of maximum egg laying) until egg laying ceased. Placement of the L4 stage hermaphrodite was scored as day 0, and the reproductive span was defined as day 0 until cessation of egg laying. Hermaphrodites were removed, and progeny (eggs or hatched larvae) on the surface or edge of the dish were manually counted. Matricidal animals were excluded from the analysis.

In the first set of experiments, one hermaphrodite was mated with two males for 48 h. However, this protocol generated hermaphrodites that were self-fertilized, partially cross-fertilized, or fully cross-fertilized. Therefore, we changed to another protocol: males used for mating were pre-labeled with a fluorescent marker by placing L4 males on NGM plates containing 1 μg/ml of red fluorescent Mitotracker CMXRos (Life Technologies) for 17 h [28–30]. A total of 15 unlabeled hermaphrodites were mated with 30 labeled males for 12 h and individual hermaphrodites were then sorted based on the presence of fluorescent sperm in the spermatheca to identify cross-fertilized hermaphrodites. Data was submitted to the non-parametric Kruskal-Wallis analysis of variance.

Developmental stage was estimated by observation of the vulva [48]. Animals were synchronized by transfer after a 2-h laying period, as described above for the lifespan analysis, and incubated at 20°C. At the L4 to adult transition, animals were washed from the plates at three time points, mounted on agarose pads, and examined using a compound microscope. Animals were visually categorized into age groups based on vulval development as described [49]. At least 40 animals per condition were scored.

*D. melanogaster*: Eggs were collected in 24-h laying periods and scored for 2-day-old and 15-day-old animals. The fecundity of the females varied on a 48-h cycle under the conditions used. Developmental time was estimated by placing 30 first instar larvae into a vial, and collecting and counting the resulting adults in 12-h windows. Five vials per condition were analyzed.

### Quantification of *C. elegans* swimming behavior

Four or five adult hermaphrodites (day 3 or day 7 of adulthood) per condition were randomly selected and deposited in 60 μl M9 buffer on a 10-mm etched circular slide (Delta Microscopies). This low density minimized overlap between worm movements and allowed each worm to be accurately tracked. Worms were allowed to acclimate for 30 s and their movements were then recorded for 45 s. Videos were processed using ImageJ software and analyzed with the CeleST program implemented in MATLAB. Eight videos per condition were realized. The recording system consisted of a Leica M205FA stereomicroscope, a charge-coupled device camera recording at 21 frames/s, and LAS AF software to control image acquisition. A minimum of 30 worms per sample was analyzed.

### Statistical analysis

Lifespan analysis and calculations were performed using Oasis online software [50] and *p*-values were calculated using the Mantel–Cox log-rank method. All other comparisons were performed using variance analysis and *t*-tests with MATLAB software. Motility data were subjected to principal component analysis and analysis of variance using MATLAB. Levene test was used to assess equality of variances. A *p* value of 0.05 was considered statistically significant. Replicate experimental data are shown in Tables 1 and 2 for *D. melanogaster* and *C. elegans*, respectively. When Kruskal-Wallis or ANOVA gave significant results, post-hoc tests (Dunnett test) handling multiple comparisons were performed.

## Supplementary Materials

Supplementary Figures

Supplementary Tables
