# Supplementary material for: Parental age effect on the longevity and healthspan in Drosophila melanogaster and Caenorhabditis elegans
Source: Aging (Albany NY). 2023 Nov 1;15(21):11720–39. doi: 10.18632/aging.205098 (PMC10683632; doi:10.18632/aging.205098)
Supplement: Supplementary Figures [file aging-15-205098-s001.pdf]

SUPPLEMENTARY FIGURES

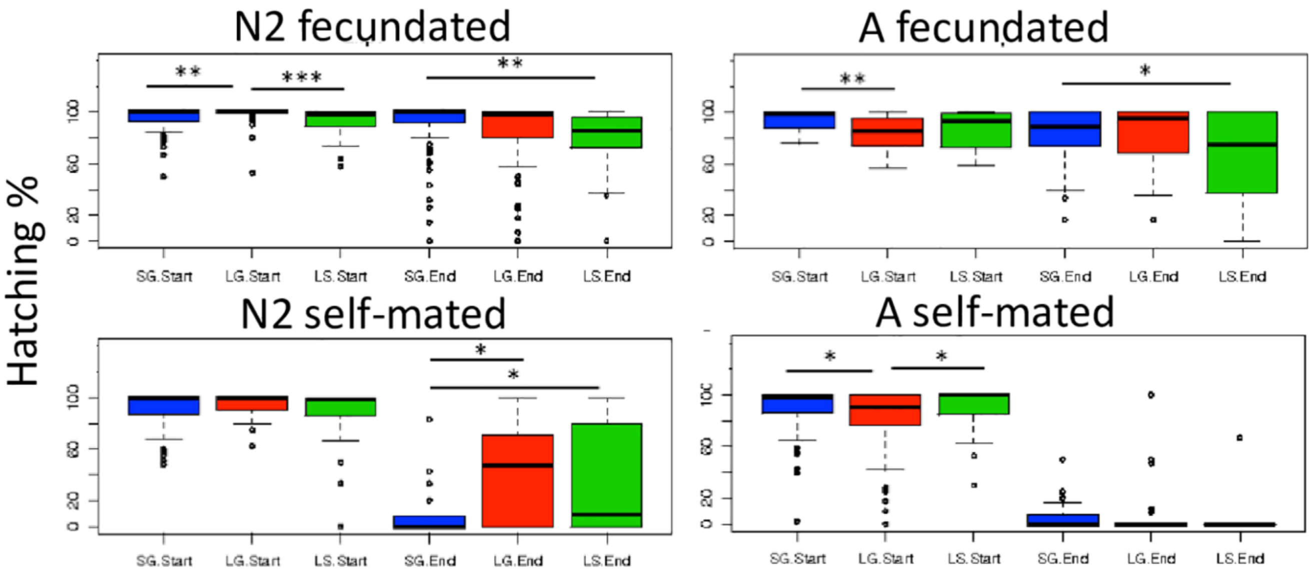

Supplementary Figure 1. Fertility of the *C. elegans* lines. Data show the hatching percentage of eggs laid by hermaphrodite progeny of four short generations (SG), two long generations (LG), or two LG followed by reversal to one SG (LS). Fertility was measured for 60 h at the beginning of the reproductive life (indicated by Start, where 0 h = plating of the L4 stage hermaphrodite) and for 36 h at the end of the reproductive life (indicated by End, 96–132 h after plating). Cross-fertilized animals maintained their fertility throughout their reproductive span, while the fertility of the self-fertilized animals was reduced during the final days of their reproductive span. Boxes, box edges, and whiskers represent the mean, IQR, and SEM, respectively, with outlier values as dots. $N > 20$ animals per condition. $^{*}p < 0.05$, $^{**}p < 0.001$, $^{***}p < 0.0001$ by pairwise *t*-test. Interactions between conditions and time have been excluded by type III two-way ANOVA.

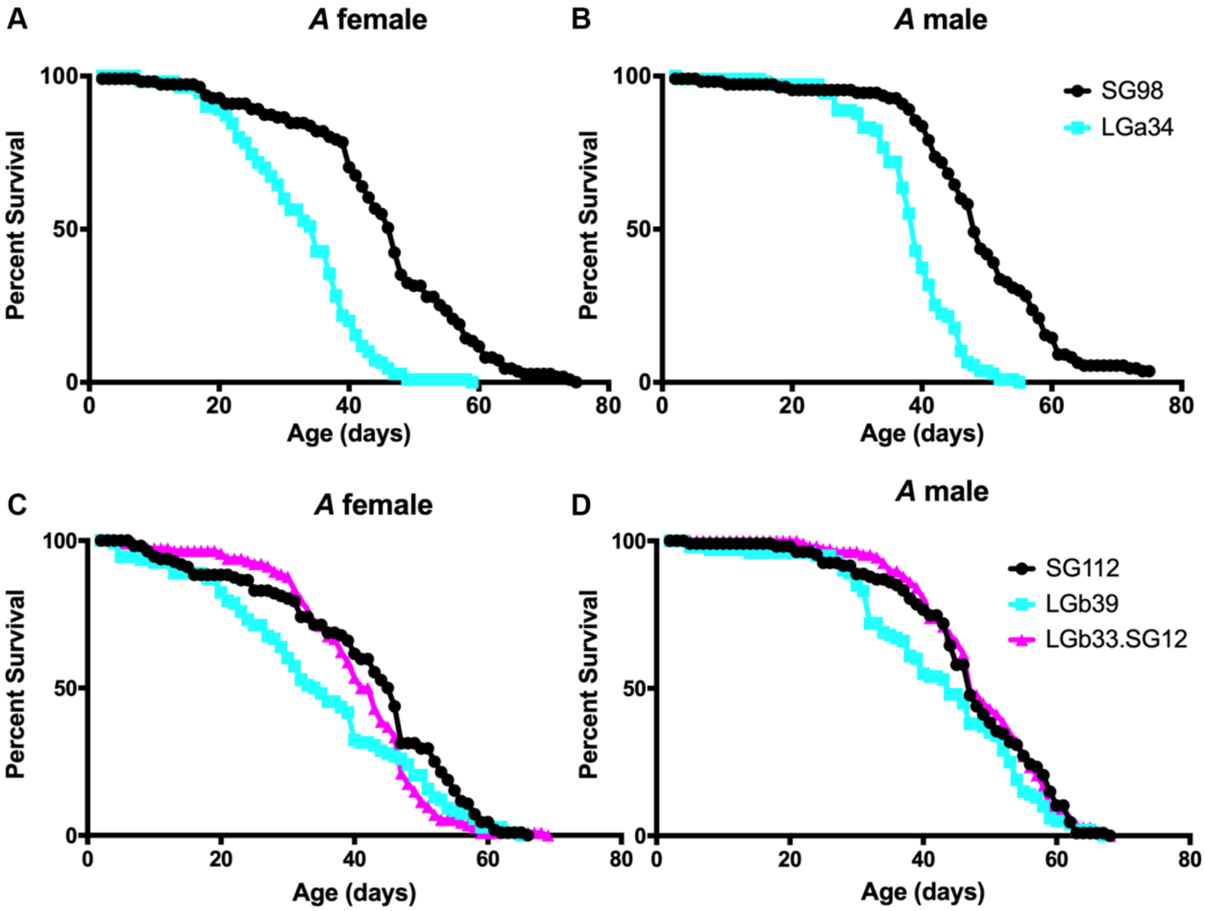

Supplementary Figure 2. Effect of parental age on the longevity of *D. melanogaster* progeny. (**A**–**D**) Lifespan analysis of female (**A**, **C**) and male (**B**, **D**) for *A* lines flies. Lifespan curves of the data presented in Table 2 are presented in this supplementary figure.

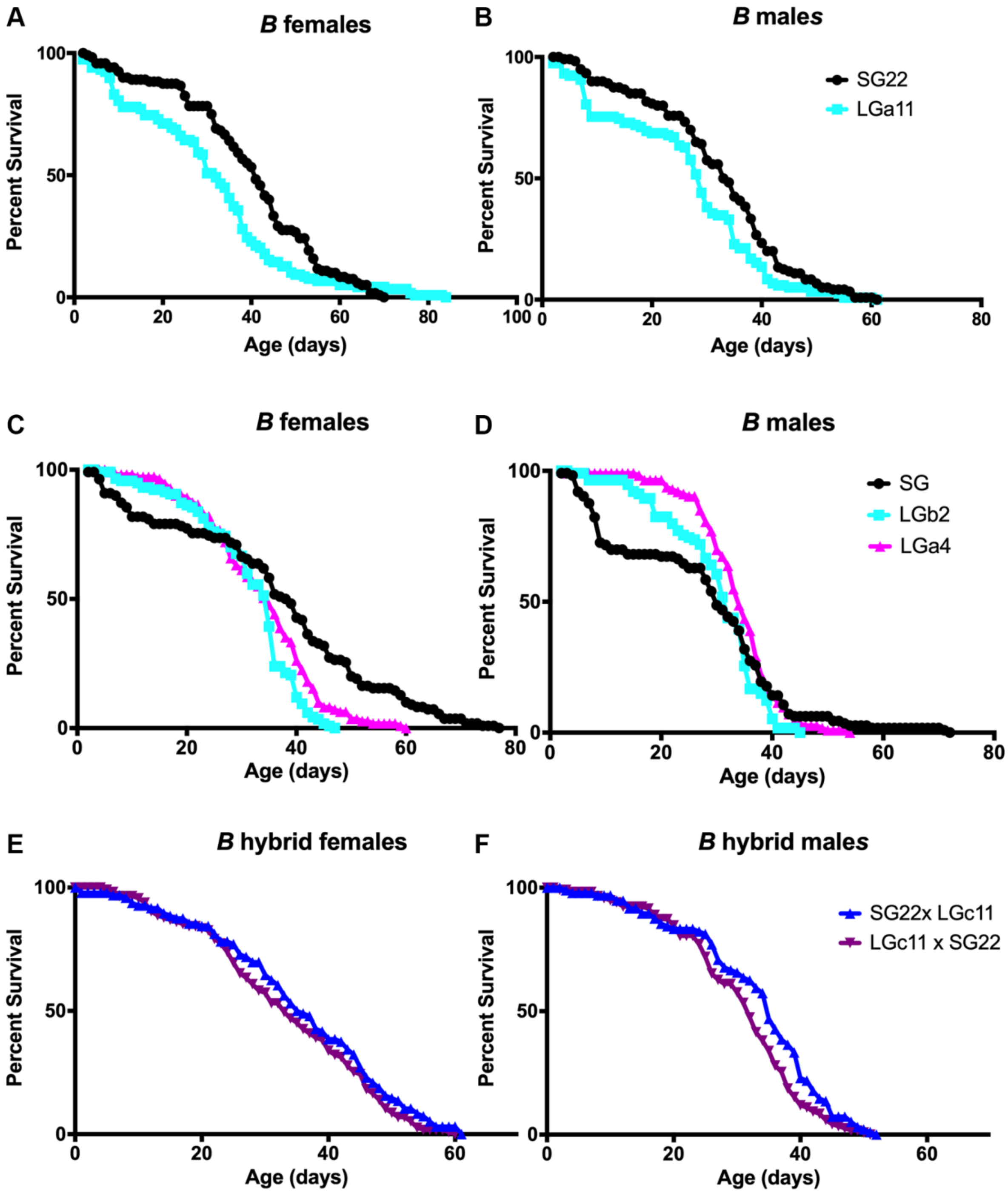

Supplementary Figure 3. Effect of parental age on the longevity of *D. melanogaster* progeny. (**A**–**F**) Lifespan analysis of female (**A**, **C**, **E**) and male (**B**, **D**, **F**) for *B* lines (**A**–**D**). (**E**, **F**) Lifespan analysis of hybrid progeny from reciprocal crosses of the indicated *B* lines. Hybrids are from females SG22 x males LGc11 or females LGc11 x males SG22. *N* = 89–119 per line. See Table 2 for replicate experiments and statistical analysis.
