# Supplementary material for: Parental age effect on the longevity and healthspan in Drosophila melanogaster and Caenorhabditis elegans
Source: Aging (Albany NY). 2023 Nov 1;15(21):11720–39. doi: 10.18632/aging.205098 (PMC10683632; doi:10.18632/aging.205098)
Supplement: Supplementary Tables [file aging-15-205098-s002.pdf]

SUPPLEMENTARY TABLES

Supplementary Table 1. Lifespans of the wild type strains used in this study.

Strain	95% C.I. (days)	Mean LS ± SE (days)	Number of animals	Trans generational experiments
CX11262	16.8 ~ 17.8	17.29 ± 0.26	332	
JU829e	16.8 ~ 18.2	17.48 ± 0.35	163	
JU1200c	13 ~ 14.2	13.57 ± 0.31	153	
CB4856	21.4 ~ 22.7	22.04 ± 0.33	357	
CB4852	18.5 ~ 19.1	18.77 ± 0.15	474	
JU775d	23.2 ~ 25.3	24.26 ± 0.56	159	
JU1088c	15.7 ~ 17	16.36 ± 0.33	256	**
JU1440c	16.4 ~ 17.6	17.01 ± 0.32	230	**
N2c	17.8 ~ 18.5	18.14 ± 0.17	667	*
JU1171e	17.7 ~ 18.6	18.18 ± 0.21	342	*
JU1652c	18.6 ~ 19.9	19.27 ± 0.33	184	*Named C
JU319b	21.6 ~ 23.4	22.48 ± 0.45	339	*Named A
JU1580f	21.4 ~ 23.4	22.39 ± 0.53	96	*Named B

Abbreviation: LS: lifespan. *Indicates lines used in transgenerational experiments. **Indicates lines in which the SG lifespans have varied over several years of analysis. Lowercase letters (a–f) indicate different isolines. For strains without isoline designations, the lifespans did not vary significantly between isolines.

Supplementary Table 2. Fecundity of the *C. elegans* progeny.

Genotype-Condition	Mating status	Brood size†	% Matricide	*N*
JU1440c-SG	mated	406.7 ± 70.2	50	10
N2c-SG	mated	305.3 ± 168.1	30	10
JU319b-SG	mated	219.5 ± 81.1	20	10
JU1200a-SG	mated	535.8 ± 112.9	23	13
N2c-SG	mated	546.9 ± 71.6	23	13
JU1580a-SG	mated	444.2 ± 97.8	23	13
**Genotype-Condition**	**Mating status**	**Brood size§**	**% Matricide**	***N***
N2c-SG4	fecundated	589 ± 87	6	30
N2c-LG2	fecundated	560 ± 99	6	30
N2c-LG2SG1	fecundated	556 ± 71	0	30
N2c-SG4	self-mated	264 ± 27	0	18
N2c-LG2	self-mated	218 ± 131	0	18
N2c-LG2SG1	self-mated	291 ± 57*	0	20
JU319b-SG4	fecundated	442 ± 79	56	30
JU319b-LG2	fecundated	380 ± 58	50	30
JU319b-LG2SG1	fecundated	311 ± 102	73	30
JU319b-SG4	self-mated	203 ± 29	10	20
JU319b-LG2	self-mated	194 ± 71	35	20
JU319b-LG2SG1	self-mated	222 ± 30	35	20

Data used to construct Figure 3 are indicated. †Brood size scored from day 0 to day 5 of adulthood. Mated hermaphrodites were crossed to males for 48 h on the first 2 days of adulthood. §Brood size scored from 36 h to 156 h of adulthood. Cross-fertilized animals were selected after mating with Mitotracker CMXRos-labeled males. Self-fertilized brood sizes were scored from 0 h to 156 h of adulthood. Values are the mean ± SD of the indicated number of animals. *$p < 0.05$ vs. the SG animals by one-way ANOVA with Kruskal-Wallis test.
